# Flow Cytometry and Molecular Techniques Could Complement Morphological Detection of Leukemic Infiltration in Ascitic Fluids: A Case Report

**DOI:** 10.3390/medicina58020264

**Published:** 2022-02-10

**Authors:** Inés Martínez-Alfonzo, Daniel Láinez-González, Laura Solán-Blanco, Aida Franganillo-Suarez, José I. Cornejo, Amanda Garcia-Lopez, Sara Martín-Herrero, Tamara Castaño-Bonilla, Rocío Salgado-Sánchez, Teresa Arquero-Portero, María J. Cortti-Ferrari, Pilar Llamas-Sillero, Juan M. Alonso-Dominguez

**Affiliations:** 1 Department of Hematology, Hospital Universitario Fundación Jiménez Díaz, 28040 Madrid, Spain; ines.martineza@quironsalud.es (I.M.-A.); laura.solan@quironsalud.es (L.S.-B.); aida.franganillo@quironsalud.es (A.F.-S.); amanda.garcia@quironsalud.es (A.G.-L.); sara.mherrero@quironsalud.es (S.M.-H.); tamara.castano@fjd.es (T.C.-B.); rocio.salgado@quironsalud.es (R.S.-S.); tarquero@quironsalud.es (T.A.-P.); mjose.cotti@quironsalud.es (M.J.C.-F.); pllamas@fjd.es (P.L.-S.); 2 Experimental Hematology, Instituto de Investigación Sanitaria Fundación Jiménez Díaz, 28040 Madrid, Spain; daniel.lainez@fjd.es; 3 Pathology Department, Hospital Universitario Fundación Jiménez Díaz, 28040 Madrid, Spain; jignacio.cornejo@quironsalud.es

**Keywords:** acute myeloid leukemia, nucleophosmin gene (*NPM1*) mutation, ascites

## Abstract

Extramedullary involvement of acute myeloid leukemia (AML) is infrequent, and ascitic infiltration is even more unusual. We present a case of a 48-year-old woman diagnosed with NPM1-mutated AML that debuted with ascites, for which morphological studies of the ascitic fluid did not detect leukemic infiltration, maybe due to technical problems in the sample preparation. Multiparameter flow cytometry (MFC) detected a blast population compatible with AML, and allele-specific PCR detected NPM1-mutated transcripts. Body fluid infiltrations are an infrequent initial manifestation or sign of progression in AML. As far as we know, this is the first reported case of an NPM1-mutated AML that debuted with ascites, and also the first description of the utilization of molecular techniques to detect the leukemic origin of the ascites. This case highlights that, given that allele-specific PCR and MFC increase the sensitivity of morphological studies, these techniques should be routinely applied in the study of any kind of effusion detected in an AML patient.

## 1. Introduction

Acute myeloid leukemia (AML) with nucleosphosmin gene mutation (*NPM1*) is currently considered a definitive entity in the revised WHO classification of 2017 [1]. Mutation of *NPM1*-mutated AML represents 25–35% of AMLs, and typically occurs in adults with a normal karyotype (45–60%) [2]. Although *NPM1* mutation confers a good prognosis, clinical outcomes also depend on concomitant FLT3-ITD mutation and its allelic ratio [3,4].

AML extramedullary involvement is infrequent and affects mainly the gums, lymph nodes, liver, spleen and skin [5]. Ascitic infiltration by AML is even more unusual. To the best of our knowledge, we report the first case of an *NPM1*-mutated AML that debuted with ascites. The morphological diagnosis was compatible with a non-malignant origin of the ascites. Nevertheless, molecular biology and multiparameter flow cytometry (MFC) techniques established the leukemic origin of the liquid.

## 2. Case Report

A 48-year-old woman with a four-month history of irritable bowel syndrome, abdominal pain, fatigue and weight loss of 10 kg, was admitted to the Emergency Department in January 2018. Physical examination revealed ascites. No adenopathies or visceromegalies were detected. Peripheral blood analysis showed a normal leukocyte count (6860 cells/uL), moderate neutropenia (600 neutrophiles/uL), mild anemia (11.2 g/dL), and thrombocytopenia (111,000 platelets/uL). A total of 7% of cellularity consisted of large blasts with basophilic cytoplasm, azurophilic granulation and lax chromatin nuclei with prominent nucleoli. Lactate dehydrogenase (LDH) levels were elevated (867 IU/L), and hypoalbuminemia was also detected (2.8 mg/dL).

A bone marrow (BM) aspirate smear showed monomorphic infiltration by myeloid blasts without significant evidence of myeloid maturation (i.e., FAB M1). MFC analysis demonstrated that most cells (75% of the total cellularity) were positive for CD117 and CD33; weak positive for CD45, CD13, CD64 and MPO; and negative for HLA-DR, CD15, CD11b, CD16, CD56, CD203c and CD2. These findings were compatible with an AML with specific markers of the promyelocyte stage. A 46,XX, inc [17] karyotype was found and the translocation t(15;17)(q24q22) *PML/RARA* was not detected by fluorescence in situ hybridization (FISH). A mutation type A in exon 12 of the *NPM1* gene was detected, and an *FLT3*-ITD mutation was not present. A 30-gene next-generation sequencing (NGS) panel, namely Myeloid Solution (Sophia Genetics, Lausanne, Switzerland) was performed. NGS analysis revealed *DNMT3* AG550R, *IDH1* R132H, *KRAS* G12D, *CSF3R* T618I, *CSF3R* S783Qfs*6 mutations, and an *NPM1* type A mutation was confirmed.

Tumor markers were requested and a CA125 elevation was evidenced (191.9 U/mL). Parasite stool tests, quantyferon determination and viral serology were negative. A thoraco-abdominopelvic computed tomography (CT) scan revealed the presence of abundant ascites and both pericardial and bilateral pleural effusion. The echocardiogram showed 60% left-ventricular ejective function (LVEF), without hemodynamic compromise. The CT scan also described thickening of the long segment of the ileum, infradiaphragmatic lymphadenopathies, signs of peritoneal carcinomatosis, and heterogeneous changes in the left ovary. Gynecologic magnetic resonance imaging showed bilateral hemorrhagic cysts and a myomatous uterus.

**Figure 1:**
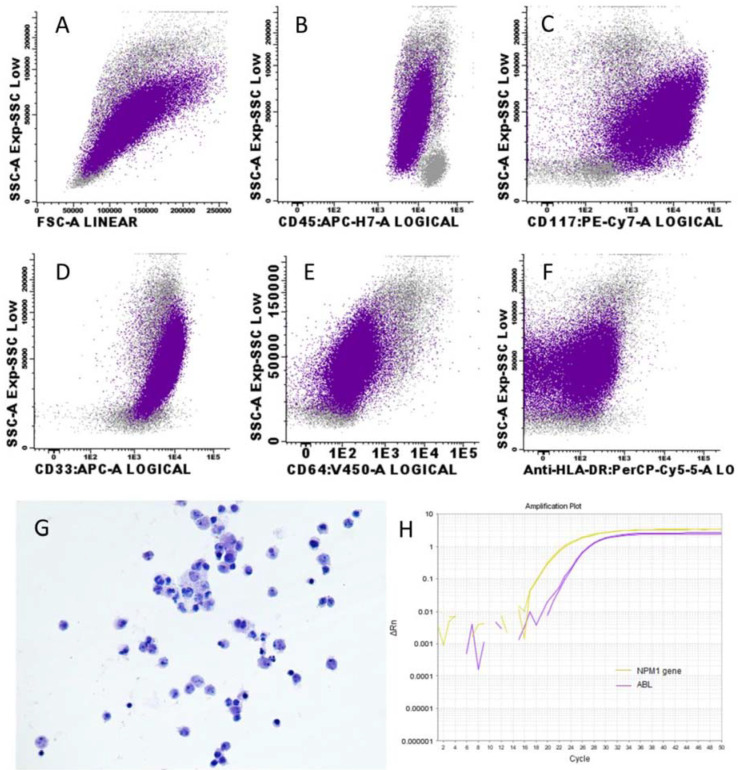
Study of ascitic fluid employing different techniques: (**A**–**F**) Blasts had high size and complexity and moderate CD45 expression. The blasts were positive for CD33 (bright), CD117, CD64 (dim) and CD13 (dim, not shown). The blasts were negative for HLA-DR and CD15 (not shown); (**G**) Ascitic fluid infiltration by mixed inflammatory cellularity, predominantly lymphocytic polymorphic appearance and scarce mesothelial epithelial cellularity; (**H**) *NPM1* mutation A-allele-specific PCR showing *NPM1* and *ABL* curves of amplification in duplicates.

The diagnostic paracentesis showed a cloudy yellowish fluid with a serum ascites albumin gradient (SAAG) < 1.1 g/dL, a leukocyte count of 4470 cells/mm^3^ with 670 neutrophils/mm^3^ and 3665 monocyte-looking cells/mm^3^. Cytologic evaluation of the ascitic fluid manifested infiltration by mixed inflammatory cellularity, predominantly lymphocytic and scarce mesothelial epithelial cellularity, suggestive of an inflammatory origin. MFC distinguished the following cell groups: neutrophils (11.53%), monocytes/histiocytes (<1%), lymphocytes (8.33%), and a population of blasts that constituted 80% of the cellularity analysed, whose abnormal phenotype has been described in BM (Figure 1). Once the diagnosis of *NPM1*+ AML debuting with ascitic fluid infiltration and probable infiltration of both pleural and pericardial fluids was established, we initiated induction treatment according to Spanish Pethema protocol with idarubicin and cytarabine (3+7); we reached complete remission (CR) with *minimal residual disease* (MRD) by MFC of 0.10% and a ratio of 0.56% by allele-specific PCR in the BM (with *ABL* employed as control gene). However, the ascites, diarrhea and abdominal pain persisted. A gastroscopy, ileoscopy and colonoscopy were performed, with biopsies confirming no underlying malignant or infiltrative digestive pathology. A new study of the ascitic fluid was performed after the administration of the induction chemotherapy, showing persistence of 8.7% of myeloid blasts with an immunophenotype similar to that described at diagnosis, and a ratio of 16.3% by *NPM1* mutation A allele-specific PCR was detected (Figure 1). Therefore, although a CR was achieved in BM, the patient showed extramedullary disease. This response should be classified as non-CR according to ELN guidelines [6].

A second echocardiogram revealed severe ventricular dysfunction (LVEF of 35%) and persistence of moderate pericardial effusion, so we decided to avoid anthracyclines and treated the patient with a cycle of high dose cytarabine (HiDAC). She reached complete cytological remission (1% blasts) in BM with MRD by MFC of 0.95%, and an *NPM1* ratio of 686%. Due to her poor performance status (i.e., ECOG 3), she initiated non-intensive treatment with 5-azacitidine, of which she only received one cycle. A few weeks later, an increase in leukocyte and blast count was documented in the peripheral blood, and a rapid increase in ascites was noted. The patient finally died from upper gastrointestinal bleeding.

## 3. Discussion and Conclusions

Our patient was diagnosed with AML with mutated *NPM1*. Cytogenetic analysis showed no alterations, and additional mutations detected by NGS did not modify the prognosis according to European LeukamiaNet (ELN) 2017 guidelines [6]. Therefore, the patient was classified as a favorable risk. In spite of the apparently favorable ELN classification, our patient had a dismal outcome due to a lack of response after the induction cycle and a worsening of her PS, which made her no longer fit for intensive chemotherapy. ELN 2017 guidelines state that only concomitant *FLT3*-ITD mutations modify the prognosis of AML with mutated *NPM1* [6]. Our patient had no *FLT3*-ITD mutation but there are additional molecular and immunophenotypic features that, although not included in ELN classification, seems to have prognostic value. A recent study reports decreased overall survival (OS) in patients with the triple combination of *NPM1*, *DNMT3A* and *IDH1* or *IDH2* mutations, as in our case [7]. A subtype of *NPM1*+ AML has been described as presenting negativity for CD34 and HLA-DR, resembling the typical immunophenotype of acute promyelocytic leukemia, as in the case reported herein. The prognostic implications of this specific immunophenotypic profile are not well known, although some studies show a greater overall survival in these patients [8]. We have no explanation for the discordant results between morphological blast count and *NPM1* quantitation in BM performed after the second cycle of chemotherapy, given that these measurements seem to be very concordant [9].

The study of ascitic fluid through paracentesis is necessary in all patients with AML, since more frequent causes of ascites should be ruled out such as liver cirrhosis, infections, hypoalbuminemia or venous thrombosis. Ascites related to malignant disease have been reported mainly in solid tumors, and as a complication of some lymphomas [10]. Extramedullary infiltration by monocytic leukemia is described in almost all body sites, including the spleen, liver, skin and lymph nodes [1,11]; however, involvement in the form of leukemic ascites as an initial or relapse manifestation of the disease is rare. In our case there was no study of pleural or pericardial fluid, but given the presentation and evolution of the disease, a leukemic origin was assumed. In a study that included 148 autopsies of patients with different types of leukemia, 49 presented ascites but none had infiltration by leukemic cells, although this could be due to the non-utilization of flow cytometry and molecular techniques [12]. Rowlands C. reported a case of leukemic ascites and jaundice as an initial presentation of granulocytic sarcoma [13]. On the other hand, Khan M et al. reported the leukemic infiltration of pericardial, pleural, and peritoneal effusions, as in our case, as manifestations of the progression from a myelodysplastic syndrome to an AML [14]. Some cases of ascitic fluid infiltration by leukemic cells have also been described as part of the initial presentation of acute myelomonoblastic leukemia, or as a late extramedullary relapse of an M4 AML after allogeneic BM transplant [15,16]. The AML of our patient showed no monocytic or myelomonocytic morphology which makes the infiltration of peritoneal fluid even more unusual.

In our case, the origin of ascites could be mixed (hypoalbuminemia due to malnutrition associated with heart failure and infiltration by AML). Morphological studies, historically employed in the diagnosis of effusions, may have a lack of sensitivity to detect malignant origin and flow cytometry, and molecular techniques should be employed. Nevertheless, the lack of detection in our case seems to be due to a technical problem with the preparation of the sample, given that ascitic fluid showed a massive leukemic infiltration by MFC. We did not perform molecular analysis of the ascitic fluid at diagnosis because we had not fully characterized the AML at that time, but we should have extracted and stored the DNA. Nevertheless, we could detect NPM1-mutated transcripts in the ascitic fluid after the first cycle of induction. Additionally, MFC and molecular techniques can provide a quantifiable parameter for assessing minimal residual disease, which could help to individualize the postremission therapy of the patients.

As far as we know, this is the first reported case of an *NPM1*-mutated AML that debuted with ascites, and also the first description of the utilization of molecular techniques to detect the leukemic origin of the ascites. This case report highlights the importance of ruling out a leukemic origin by sensitive techniques of any effusion that patients with AML present at diagnosis, or during disease evolution.

## Data Availability

Not applicable.

## References

[1] Arber D.A., Orazi A., Hasserjian R., Thiele J., Borowitz M.J., Le Beau M.M., Bloomfield C.D., Cazzola M., Vardiman J.W. (2016). The 2016 revision to the World Health Organization classification of myeloid neoplasms and acute leukemia. Blood.

[2] Döhner H., Weisdorf D.J., Bloomfield C.D. (2015). Acute Myeloid Leukemia. N. Engl. J. Med..

[3] Papaemmanuil E., Gerstung M., Bullinger L., Gaidzik V.I., Paschka P., Roberts N.D., Potter N.E., Heuser M., Thol F., Bolli N. (2016). Genomic Classification and Prognosis in Acute Myeloid Leukemia. N. Engl. J. Med..

[4] De Kouchkovsky I., Abdul-Hay M. (2016). Acute myeloid leukemia: A comprehensive review and 2016 update. Blood Cancer J..

[5] Liesveld J.L., Lichtman M.A., Lichtman M.A., Kipps T.J., Seligsohn U., Kaushansky K., Prchal J.T. (2016). Acute Myelogenous Leukemia. Williams Hematology.

[6] Döhner H., Estey E., Grimwade D., Amadori S., Appelbaum F.R., Büchner T., Dombret H., Ebert B.L., Fenaux P., Larson R.A. (2017). Diagnosis and management of AML in adults: 2017 ELN recommendations from an international expert panel. Blood.

[7] Dunlap J.B., Leonard J., Rosenberg M., Cook R., Press R., Fan G., Raess P.W., Druker B.J., Traer E. (2019). The combination of NPM1, DNMT3A, and IDH1/2 mutations leads to inferior overall survival in AML. Am. J. Hematol..

[8] Mason E.F., Kuo F.C., Hasserjian R.P., Seegmiller A.C., Pozdnyakova O. (2018). A distinct immunophenotype identifies a subset of NPM1-mutated AML with TET2 or IDH1/2 mutations and improved outcome. Am. J. Hematol..

[9] Jo S.Y., Park S.H., Kim I.S., Yi J., Kim H.H., Chang C.L., Lee E.Y., Cho Y.U., Jang S., Park C.J. (2016). Correlation of NPM1 Type A Mutation Burden With Clinical Status and Outcomes in Acute Myeloid Leukemia Patients With Mutated NPM1 Type A. Ann. Lab. Med..

[10] Smith E.M., Jayson G.C. (2003). The current and future management of malignant ascites. Clin. Oncol..

[11] Campidelli C., Agostinelli C., Stitson R., Pileri S.A. (2009). Myeloid sarcoma: Extramedullary manifestation of myeloid disorders. Am. J. Clin. Pathol..

[12] Prolla J.C., Kirsner J.B. (1964). The Gastrointestinal Lesions and Complications of the Leukemias. Ann. Intern. Med..

[13] Rowlands C.G. (1999). Cytology of ascitic fluid in a patient with granulocytic sarcoma (extramedullary myeloid tumor). A case report. Acta Cytol..

[14] Khan M.Y., Hussein K.K., Walter M.G., Hasan M.K., Kern W., Kharfan-Dabaja M.A. (2004). Granulocytic sarcoma presenting with malignant anasarca in a patient with secondary acute myeloid leukemia. Int. J. Hematol..

[15] Pantanowitz L., Steingart R., Miller K.B., Kruskal J.B., Pihan G. (2005). Leukemic ascites. Arch. Pathol. Lab. Med..

[16] Simel D.L., Weinberg J.B. (1985). Leukemic ascites complicating acute myelomonoblastic leukemia. Arch. Pathol. Lab. Med..

